# Clinical Risk Management: As Modern Tool for Prevention and Management of Care and Prevention Occupational Risk

**DOI:** 10.3390/ijerph19020831

**Published:** 2022-01-12

**Authors:** Raffaele La Russa, Stefano Ferracuti

**Affiliations:** 1 Department of Clinical and Experimental Medicine, University of Foggia, 71122 Foggia, Italy; 2 Department of Human Neuroscience, Sapienza University, Piazzale Aldo Moro 5, 00165 Rome, Italy; Stefano.ferracuti@uniroma1.it

Clinical Risk Management aims to improve the performance quality of healthcare services through procedures that identify and prevent circumstances that could expose both the patient and the healthcare personnel to risk of an adverse event.

This consists of perceiving future problems and changes in advance, in order to effectively plan the consequent actions.

In healthcare systems, where organizational dynamics and technological evolution are constantly changing, risk management must constantly assess whether these changes may lead to new opportunities for errors and new risks of adverse events that could result in to healthcare litigations.

In the study by La Russa et al. [1], a retrospective analysis of civil litigation of the Sant’ Andrea Hospital in Rome, 40% of total litigation in the five years of 2012–2016 involved the orthopedics, traumatology, emergency, general surgery, neurosurgery, and radiology departments. Especially in these wards, medical practice quality should be improved, and a clinical risk management specialist should be employed to manage malpractice claims.

Considering morgue and necropsy activity, as explained in the study performed by Del Fante et al. [2], operators of necropsy services are subjected to potential dangers or threats such as the postural risk for manual handling of loads and noise or vibration exposure.

Tomao et al. [3], instead, analyzed biological risks related to necropsy activities, which can expose individuals to infectious diseases directly (e.g., accidental punctures or wounds and splashes of biological materials) or indirectly (e.g., inhalation of aerosol particles). This requires an attentive risk analysis, through environmental and air microbiological monitoring, clinical–anamnestic questionnaires where the operators are questioned on if they use personal protective equipment (PPE), the adoption of specific prevention measures, and the performing of bacteriological and virological tests on cadaveric samples.

The results of these surveys have highlighted the importance of accurate identification of hazards, the assessment of exposure methods, and the adoption of risk-limiting measures and operational preventive protocol.

Clinical risk management is involved in all healthcare activities, and when an adverse event occurs it is essential to be able to learn from it. In these cases, it is necessary to identify not the culprits but the causes.

This is what happens, for example, in the case of nosocomial infections. In the study by Bolcato et al. [4], the case of Mycobacterium Chimaera is reported, a bacterium whose infection is characterized by specific signs and specific symptoms (e.g., emboli on cardiac valves, neurological, ocular, and auditory damages) that usually manifest after 20 months. This bacterium disseminates through aerosol mechanisms, and is frequent in patients who undergo heart surgery with exposure to contaminated heater–cooler units (HCU). For this reason, cardiologists and cardiac surgeons must nowadays perform follow-ups and prevention in cardiac surgery patients.

Clinical risk management also has to provide guidelines in case of emergencies, such as acute drug intoxication or Fatal Foreign Body Aspiration (FBA). In the first case, a study by Piccioni et al. [5] highlights how drug abuse is an increasing phenomenon among young people. The identification of a standardized treatment protocol is important for healthcare workers to manage intoxicated patients and choose between hospitalization, discharging, or temporary observation units (TUOs).

The second case, described in the study by Montana et al. [6], represents pediatric emergency, which is considered the fourth cause of accidental death in children. For this reason, it is recommended that everyone learns the Heimlich maneuver, especially teachers and childcare providers, and parents should be conscious of the risks associated with eating some solid food (e.g., sausages) and the importance of constant supervision, even during playing.

Finally, the pandemic spread of COVID-19 represents a new challenge for risk management.

The study by Zanza et al. [7] focuses on the hypercoagulable state induced by COVID-19 infection, which increases the risk for venous thromboembolism and consequently makes thromboprophylaxis mandatory unless contraindicated.

However, risk management in the COVID-19 era involves not only healthcare structures but even necropsy activities (Tomao et al. [3]), penitentiary facilities (Pagano et al. [8]), and nursing homes (Bolcato et al. [9]). 

In all these cases, a preventive screening protocol was drawn up in order to contain infection risks and provide, especially in nursing homes, the possibility for the residents to safely interact, through digital interactions or distanced visits, with their family members.

## Data Availability

Not applicable.
